# Mechanobiology Platform Realized Using Photomechanical Mxene Nanocomposites: Bilayer Photoactuator Design and In Vitro Mechanical Forces Stimulation

**DOI:** 10.3390/ma15196869

**Published:** 2022-10-03

**Authors:** Dong Niu, Yanli Zhang, Jinlan Chen, Dachao Li, Chunmeng He, Hongzhong Liu

**Affiliations:** 1 State Key Laboratory for Manufacturing Systems Engineering, Xi’an Jiaotong University, Xi’an 710049, China; 2 Key Laboratory for Molecular Genetic Mechanisms and Intervention Research on High Altitude Disease of Tibet Autonomous Region, Medical College, Xizang Minzu University, Xianyang 712082, China; 3 The Joint Key Laboratory of Graphene, Xi’an Jiaotong University, Xi’an 710049, China

**Keywords:** mechanotransduction, extracellular force loading, photomechanical soft bilayer, Mxene, controllability, adjustability

## Abstract

Mechanotransduction is the process by which cells convert external forces and physical constraints into biochemical signals that control several aspects of cellular behavior. A number of approaches have been proposed to investigate the mechanisms of mechanotransduction; however, it remains a great challenge to develop a platform for dynamic multivariate mechanical stimulation of single cells and small colonies of cells. In this study, we combined polydimethylsiloxane (PDMS) and PDMS/Mxene nanoplatelets (MNPs) to construct a soft bilayer nanocomposite for extracellular mechanical stimulation. Fast backlash actuation of the bilayer as a result of near-infrared irradiation caused mechanical force stimulation of cells in a controllable manner. The excellent controllability of the light intensity and frequency allowed backlash bending acceleration and frequency to be manipulated. As gastric gland carcinoma cell line MKN-45 was the research subject, mechanical force loading conditions could trigger apoptosis of the cells in a stimulation duration time-dependent manner. Cell apoptotic rates were positively related to the duration time. In the case of 6 min mechanical force loading, apoptotic cell percentage rose to 34.46% from 5.5% of the control. This approach helps apply extracellular mechanical forces, even with predesigned loading cycles, and provides a solution to study cell mechanotransduction in complex force conditions. It is also a promising therapeutic technique for combining physical therapy and biomechanics.

## 1. Introduction

Mechanotransduction is the mechanism through which cells sense and transduce mechanical forces into biological signals [1,2,3,4]. It plays a vital role in a number of biological processes, including gene expression [5,6], differentiation [7,8], migration [9,10], and metabolism [11,12]. A systematic study on cell mechanotransduction is now being conducted in an in vitro setting, imitating the effects of gravity [13,14], fluid shear stress [15,16,17], compressive and stretching mechanical loads [18,19,20,21], and biomedical forces that are applied to cells using external loading conditions. In vitro actuation methods, such as flow-chamber-induced fluid shear stress and stretching or tensile and compressive conditions induced by deformable membranes subjected to electromagnetic or pneumatic fields, have been meticulously designed to simulate the mechanical stimulation of living cells in vivo [18,22,23,24,25,26]. However, these methods either measure the average response of hundreds of thousands of cells over a range of centimeters or are incapable of simulating the temporal fluctuations that occur in actual biological settings. Therefore, there is a need to design technology that is capable of mechanically actuating individual cells or small colonies of cells and that can apply pressure that changes over time in a well-contained and regulated manner.

Some strategies have allowed interactions between isolated cells and realistic and complex biological environments to be studied, such as microfluidic devices [27,28,29,30], microelectromechanical systems, and other microfabrication approaches, such as micromagnetic and electrostatic approaches [31,32,33,34,35,36,37]. Although individual cells have been mechanically stimulated using these loading approaches, the absence of temporal control (microfluidic) and concerns around biocompatibility have hampered the application of these force-loading approaches. Recent advances in materials chemistry and engineering have provided researchers with stimuli-responsive materials. These materials can be used as tools to mimic the complex characteristics of natural extracellular forces. Such materials include shape memory polymers [38,39,40], liquid crystal elastomers (LCEs) [41,42], hydrogels [43,44,45], dielectric elastomers [46,47], and electroactive polymers [48,49,50]. However, using ultraviolet light to activate the mechanical motion of LCEs and some hydrogels can harm cells, limiting the applicability of these techniques. In addition, given the temporal and spatial modulation and force control issues, designing stimuli-responsive materials compliant with the multivariate mechanical stimulation of individual cells is a formidable task.

**Figure 1:**
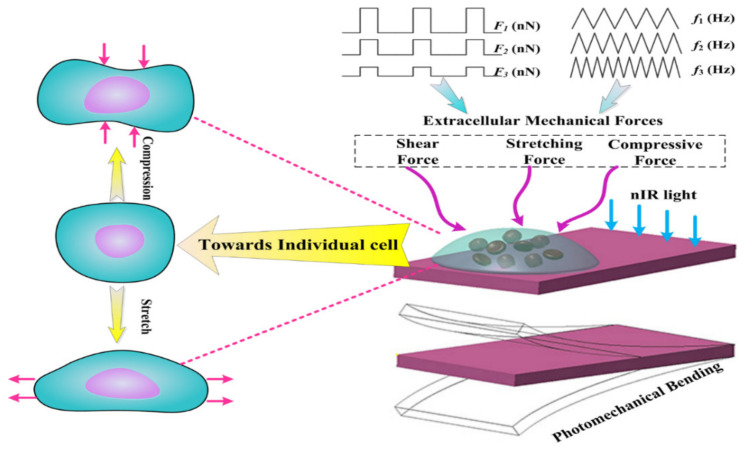
Design schemes for the mechanobiology platform realized by photomechanical soft actuators.

To overcome this issue, we designed an in vitro mechanical loading tool using MNP-based polymer nanocomposites. This bilayer consists of a pure PDMS layer and a PDMS/MNPs composite layer. Specifically, Mxene nanoplatelets (MNPs) are dispersed inside polydimethylsiloxane (PDMS) matrices to produce polymer nanocomposites. This bilayer structure was inspired by the folding phenomenon in which two sheet-like components with different mechanical properties attain a shape that facilitates an equilibrium between their constituent elements. With this tool, as depicted in Figure 1, photoresponsive and multivariate mechanical stimulation are merged to impose rapid and controlled mechanical force loading (at the tens of nN level) on individual cells or small colonies of cells. The bilayer structure allows the realization of temporal and spatial modulation as part of this new mechanobiological platform. Meanwhile, the microfabrication technique enables the dimensions to be reduced to a size that is relevant for the investigation of mechanotransduction in individual cells and small cell colonies.

## 2. Materials and Methods

### 2.1. Fabrication of the Bilayer Structure

Ti3C2Tx Mxene Nanoplatelets (MNPs, purity > 70%) with 50–150 nm thickness and 2–10 μm diameter were purchased from *Nanjing XFNANO Materials Tech Co. Ltd*. (Nanjing, China) and were directly used in their original form. Polydimethylsiloxane (PDMS, Sylgard 184 Silicone Elastomer) was used as the host matrix with the base agent and curing agent obtained from Dow Corning Corporation (Midland, MI, USA). The PDMS/MNPs composite was prepared by weighing the desired amount of MNPs and adding them to the PDMS crosslinker. Then, the PDMS base compound was added at a ratio of 10:1 to the PDMS crosslinker and mixed. The pure PDMS solution was configured using the PDMS base compound and the crosslinker at a ratio of 10:1. The fabrication procedure was facile and scalable. It only involved scrape coating and spin coating for the bottom PDMS/MNPs nanocomposite layer (800 rpm, 30 s, 80 μm in thickness) and the upper pristine PDMS layer (1600 rpm, 30 s, 50 μm in thickness), which were measured using an optical microscope (*Leica DM3 XL*, Wetzlar, Germany).

### 2.2. Observation and Characterization of Photomechanical Actuation

The MNP concentrations used in this study ranged from 1 to 5 wt%. All samples were made into strips with dimensions of 12 × 3 mm (length × width). An nIR light source with a wavelength of 808 nm was chosen to actuate the soft bilayer actuator. A *KEYENCE* displacement sensor (LK-H050, Shanghai, KEYENCE (CN) Ltd., measurement range ± 10 mm, linear accuracy 0.02% F.S.) was used to record the tip deflection of soft bilayers when the nIR light was incident from the PDMS/MNPs layer. The photomechanical bending process of the soft bilayers was experimentally measured in the case of nIR light intensity ranging from 0 to ~30 mW∙mm^−2^. Additionally, a programmable optical shutter was utilized to modulate illumination frequency, and then the displacement sensor recorded the photomechanical bending process. Meanwhile, a handmade polystyrene foam sphere (*d* = 2 mm) was chosen to demonstrate the mechanical oscillation induced by the two-step photomechanical bending of soft bilayers.

### 2.3. In Vitro Cell Mechanical Force Loading

The left side of the fabricated soft bilayers, 3 × 3 mm (length × width), was anchored at the K9 glass bases with PDMS solution as an adhesive layer, in which the width of glass bases is slightly larger than that of soft bilayers. In this case, it was helpful for constructing a cantilever actuator, which was chosen as the matrix for culturing cells onto the PDMS surface. The cell-culture medium of the gastric gland carcinoma cell line MKN-45 (ATCC, Manassas, VA, USA) was plated in 6-well culture plates at a density of 2 × 10^5^ cells per well, in which a 12 × 3 mm soft bilayer structure with 4 wt% MNP concentration was in each well. The cultured cells were allowed to grow and adhere to the thin PDMS layer by incubating them in Dulbecco’s modified Eagle medium (BI, Duluth, GA, USA) supplemented with 10% Fetal Bovine Serum (FBS, Gibco, Waltham, MA, USA) and 10% penicillin and streptomycin at 37 °C for 24 h in an incubator (JCS0084, Thermo Fish) containing 5% CO_2_. Afterwards, each soft bilayer was carefully taken out to conduct the actuation process. An nIR light (working frequency with 1 Hz) with a light intensity of 15 mW∙mm^−2^ was applied to the left side of the soft bilayer at the actuating time from 0 to 6 min in a 2 min interval. The soft bilayer with cultured cells was then put back into the incubator and cultured from 24 to 72 h.

### 2.4. Cell Counting and Cell Apoptosis Evaluation

Soft bilayers without mechanical force loading were set as the control group, while the soft bilayers with mechanical force loading were set as the experimental group. An optical microscope was utilized to record the cell growth states of the control group at 24, 48, and 72 h. The statistics of living cells were conducted based on commercial cell counting boards. Meanwhile, the flow cytometry characterizing apoptosis rates of the experimental group was determined. An Annexin V-binding assay was conducted by using an Annexin V-FITC/PI Apoptosis Detection Kit. A flow cytometer (Milema Beckman Coulter, Brea, CA, USA) was used to analyze the expression of Annexin V-FITC+/PI− (early apoptosis) and AnnexinV- FITC+/PI+ (late apoptosis) cells.

## 3. Results and Discussion

### 3.1. Two-Step Photomechanical Bending Process

**Figure 2:**
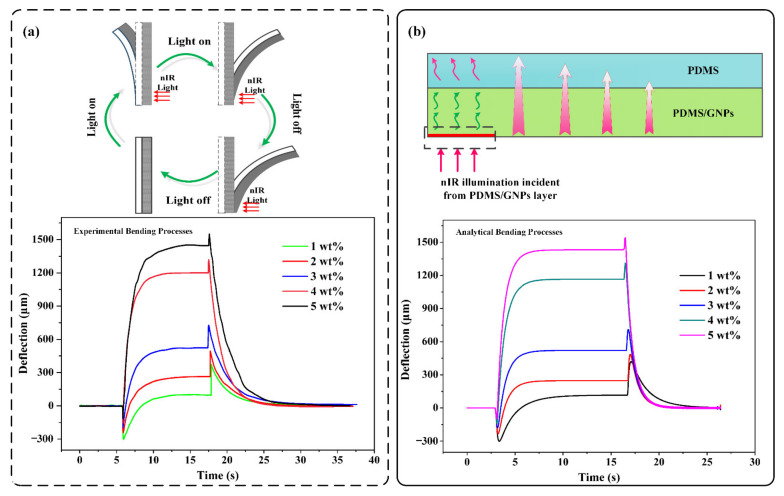
Two-step photomechanical bending of soft bilayers. (**a**) Experimental measurement of photomechanical bending of the soft bilayer. (**b**) Simulation of the two-step photomechanical bending with the help of COMSOL Multiphysics 5.0.

The strategy used to produce this soft bilayer structure was based on a straightforward layer-by-layer manufacturing technique. This technique is straightforward and scalable, and it requires only scrape-coating and spin-coating operations for the bottom PDMS/MNPs nanocomposite layer and the top pure PDMS layer, respectively. Mxene acts as a heat source when dispersed in PDMS matrices owing to its remarkable photothermal conversion efficiency in the nIR band. Meanwhile, the relevant properties of nanocomposites containing MNPs are altered as a result of the exceptional thermal conductivity and negative coefficient of thermal expansion of MNPs, which result in photomechanical actuation due to mismatch expansion between the PDMS layer and the PDMS/MNPs layer. As shown in Figure 2a, when light is incident from the PDMS/MNPs layer, a two-step bending process is observed, in which the soft bilayer bends rapidly toward the PDMS side by a small amount and then reverses to bend toward the PDMS side when the nIR light is switched on. Following the cessation of nIR light irradiation, the PDMS/MNPs layer undergoes rapid and modest bending, followed by a reversible deflection to the side of the PDMS layer. The two-step bending process includes an unusual bending process, which is fast and involves bending toward the PDMS layer when the nIR light is switched on and bending continuously toward the PDMS/MNPs layer when the nIR light is switched off, and the larger bending process, which occurs toward the PDMS layer and is determined by the bilayer effect, which slows gradually. The photomechanical backlash deflection decreases as the MNP concentration increases. In addition, when the MNP concentration increases, the terminal photomechanical deflection increases and reaches a maximum at a concentration of 5%.

To illustrate the photomechanical bending mechanism, Figure 2b depicts the bilayer photoresponsive structure, which is composed of a thin layer of PDMS/MNPs and a thin layer of PDMS that is attached and confined to the left side. When the nIR irradiation (30 mW∙mm^−2^) is incident from the pure PDMS/MNPs layer, an analogous heat source is exerted at the rear of the PDMS/MNPs nanocomposite layer because the nIR irradiation is regarded as a power source that heats up a restricted region. Heat is transferred from the rear surface of the PDMS/MNPs nanocomposite layer to the top surface of the PDMS layer along the thickness direction through the bilayer actuator. Thus, bending occurs due to differences in thermal expansion between the two thin layers. Using COMSOL Multiphysics software, the two-step analytical bending process is derived by combining the heat transport and thermal expansion processes. When comparing the analytical deflection processes with the experimental results in Figure 2a, a nearly identical bending process can be observed, which implies the effectiveness of our proposed simulation methods.

**Figure 3:**
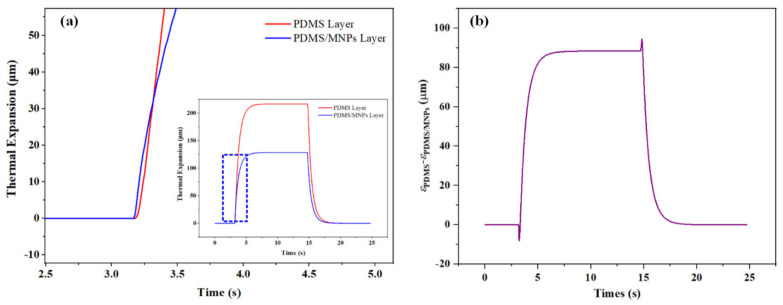
Two-step bending process analysis. (**a**) Thermal expansion curves of the PDMS layer and the PDMS/MNPs layer. (**b**) Differences in thermal expansion of these two layers for demonstrating the two-step bending process. It is not the actual photomechanical deflection, instead a kind of relative analysis.

To further explain the two-step photomechanical bending process, the individual thermal expansion process of PDMS and the PDMS/MNPSs layer was calculated with the help of COMSOL Multiphysics, as shown in Figure 3a. To demonstrate this issue concretely, the soft bilayer with 4 wt% MNPs was exemplified here. As shown in Figure 3a, we can notice that the thermal expansion of the PDMS/MNPs layer was larger than that of the PDMS layer after nIR light was applied, although the coefficient of thermal expansion for the PDMS/MNPs layer is lower. This can be attributed to the fact that an unneglected temperature gradient along the thickness of the soft bilayers existed, in which the temperature of the PDMS/MNPs layer was much higher than that of the PDMS layer. It was why the soft bilayers showed backlash bending towards the PDMS layer when the nIR light was on. Afterward, the thermal expansion of the PDMS layer started to exceed the PDMS/MNPs layer, which gave rise to another slow photomechanical bending due to the commonly reported bilayer effect. This analysis was conducted based on the core principles in the *Timoshenko* thermoelastic model, in which the two bonded layers had a mismatch in elongation that caused deflection under heat stimulus. To further explain the photomechanical bending mechanism, a simple method was utilized in our analysis only by calculating the deviation of thermal expansion, i.e., *ε_PDMS_*-*ε_PDMS/GNPs_.* As shown in Figure 3b, the curve did not show the actual deflections of the soft bilayer but was only used to depict the variation for thermal expansion. It was found that the PDMS/MNPs layer thermal expansion was larger than that of the PDMS layer. In this case, the soft bilayer photomechanically bent towards the PDMS/MNPs layer and gave rise to backlash deflection at the beginning of the actuation process. Then, PDMS thermal expansion gradually exceeded the PDMS/MNPs layer and the soft bilayer showed photomechanical bending towards the PDMS/MNPs layer and reached the final and steady deflection. Furthermore, the deviation between these two layers became larger when the nIR light was turned off and the soft bilayer experienced another backlash deflection. As time went on, the thermal expansion of each layer gradually decreased, which allowed the photomechanical bending to disappear and the soft bilayer to recover its initial state. Although in Figure 3b the actual photomechanical deflection could not be calculated, it helped to clarify the two-step photomechanical bending of our proposed soft bilayer. In a word, each layer’s unique thermal expansion process gave rise to the backlash deflection during the two-step photomechanical bending process.

### 3.2. Mechanical Forces Loading Analysis

**Figure 4:**
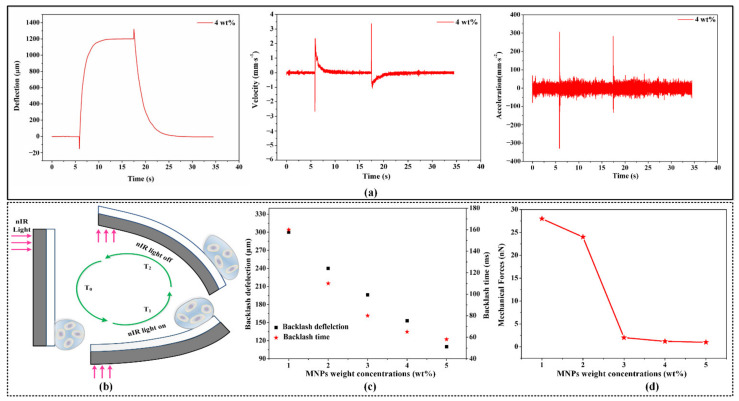
Analysis of mechanical force loading on cells based on velocity and acceleration curves. (**a**) Velocity and acceleration curves of the two-step photomechanical bending process. (**b**) Diagram of the soft bilayer two-step photomechanical-bending-induced mechanical force loading. (**c**) Backlash bending comparisons under various MNP weight concentrations. (**d**) Theoretical extracellular mechanical force exerted on cells.

Initially, a bilayer structure containing 4 wt% MNPs was chosen as the cell force loading mechanism. Given the two-step bending process in response to nIR light stimulation, it was possible to obtain velocity and acceleration curves as a function of time. As shown in Figure 4a, dramatic and rapid variations in velocity and acceleration were observed during the fast-bending process when the nIR light was switched on or off, but the velocity and acceleration varied gradually during the bilayer bending process. Moreover, the maximum velocity and acceleration (−300–300 mm∙s^−2^) occurred at a particular moment in the rapid bending process. After the cells had adhered to the surface of the bilayer, it was permissible to apply mechanical stimulation (force) to the cells due to the constant and rapid change in velocity and acceleration in accordance with Newton’s second law of motion (***F = m × a***), as shown in Figure 4b.

In addition, mechanical force loading instruments containing varying MNP concentrations were built and evaluated. As shown in Figure 4c, as the MNP concentration increased, the rapid bending process was enhanced, and the backlash deflection and time decreased significantly. Even though photomechanical deflection varied with MNPs concentration, the two-step bending process still occurred, leading to dramatic changes in velocity and acceleration and mechanical stimulation of cells from the outside. Then, the forces imposed on the cells by our bilayer force loading tool were computed based on a large cell (*r* = 100 μm), given the objective to develop a force loading tool that is applicable to single cells and small colonies of cells. Due to the light weight of single cells and small cell colonies, it is plausible to equate the acceleration of the bilayer to that of the cell itself. As shown in Figure 4d, the maximum mechanical force (~30 nN) imposed on the cell decreased as the MNP concentration increased, which was attributable to progressive slowing of the change in acceleration during the rapid bending process when the nIR light was switched on or off. The simple fabrication of this bilayer enabled the development of a cell-scale platform suitable for single-cell mechanical stimulation.

### 3.3. Mechanical Forces Adjustment with nIR Light

**Figure 5:**
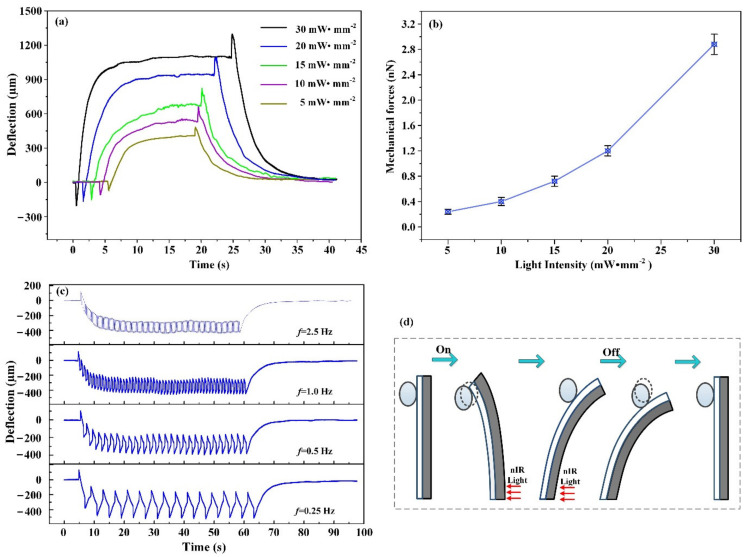
Mechanical force loading was controlled and adjusted. (**a**) Photomechanical bending was controlled by nIR light intensities ranging from 5 to 30 mW∙mm^−2^ for a bilayer containing 4 wt% MNPs. (**b**) Theoretical mechanical forces controlled by light intensities. (**c**) nIR light frequencies adjusted photomechanical bending when nIR light intensity was 15 mW∙mm^−2^. (**d**) PS microsphere oscillation under nIR light mimics the cell actuated by photomechanical actuators.

As a result of the controllability and modulation of the nIR light source, it was anticipated that our mechanical cell loading tool would be capable of temporal and spatial modulation, as well as controllable deflection. First, the photomechanical bending mechanisms under different intensities of nIR light were investigated. As shown in Figure 5a, the bending curves were measured and compared at different nIR light intensities ranging from 5 to 30 mW∙mm^−2^ for a bilayer containing 4 wt% MNPs. All the curves had almost identical bending tendencies, with the exception of the backlash and terminal deflections. In accordance with the analysis shown in Figure 3a, the fast bending process generated the force loading conditions. Then, the backlash deflections were compared, which revealed a progressive increase in photomechanical deflection as the intensity of nIR light increased. As shown in Figure 5b, the results show that when the intensity of nIR light increased, the ability of the bilayer to apply mechanical stress on cells increased. In this case, the parameter influencing photomechanical bending was only the nIR light intensity, because the coefficient of thermal expansion of the PDMS and PDMS/MNPs layer was unchanged. Increasing the nIR light intensity enlarged not only the final photomechanical deflection, but also the backlash deflection. Additionally, a series of nIR illuminations with varying frequencies was used to investigate the multicycle photomechanical actuations of the bilayer. Figure 5c shows that each method exhibited multicycle photomechanical bending with high reproducibility. When the frequency of nIR light increased (when nIR light intensity was 15 mW∙mm^−2^), the amplitude of photomechanical bending increased steadily. Adjusting the operating frequency of nIR light enables programmable modulation of photomechanical actuations in bilayer structures. Thus, it is the benefits of nIR light stimulation that enable bilayer structures to achieve programmable photomechanical deflection, in turn enabling temporal and spatial modulation of force loading on cells.

To demonstrate the applicability of our bilayer to cell mechanobiology, tests to simulate the circumstances of cell loading were performed. The photomechanical bilayer was used to stimulate a polystyrene sphere, which represented a small colony of cells. Figure 5d shows that the polystyrene sphere was suspended close to the bilayer, which had an MNP concentration of 4 wt%. The surface capillary connection between the polystyrene sphere and the PDMS layer mimics the cultivation of cells on membranes. During full photomechanical actuation of the bilayer, the polystyrene sphere is activated by the deflection process of the bilayer actuators. In accordance with the rapid bending process of the bilayer actuators, a particularly significant amplitude was observed when the nIR light was switched on and off. Higher pressures were imposed immediately on the polystyrene sphere when the nIR light was adjusted instantly, but the other deflection technique generated a sluggish force loading state owing to the gradual decrease in deflection speed. In addition, a modulated light source was used to illuminate the bilayer to generate significant amplitude deflection. As demonstrated in Video S1, the polystyrene sphere exhibited rapid and reversible oscillations. With varied modulation of the nIR light source, oscillations with unique amplitudes and frequencies were observed, which further supported the temporal and spatial controllability of the modulation. These findings suggest that the bilayer can be used as a tool to study cellular mechanotransduction after mechanical stimulation.

### 3.4. Cell Viability under Photomechanical Actuations

**Figure 6:**
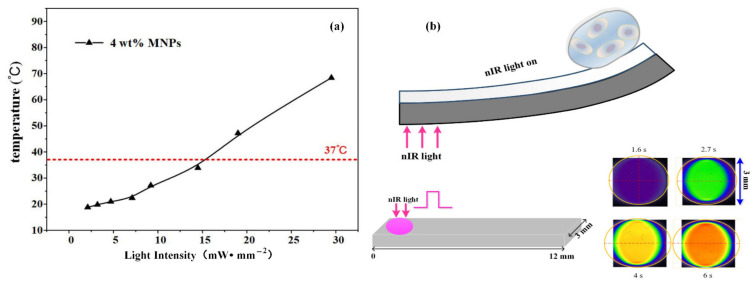
Temperature change and distribution of soft bilayers as illuminated by nIR light. (**a**) The temperature change in the soft bilayer surface under various nIR light intensities. (**b**) Temperature distribution of the soft bilayer surface.

Gastric cancer cells (MKN-45) were examined in terms of their growth and differentiation when cultured on the soft bilayer (12 × 3 mm) with 4 wt% MNPs. This analysis was performed to determine whether the mechanical forces generated in the photomechanical bending process of bilayer were physically transduced to the cells cultured on them. With an actuation time ranging from 2 to 6 min, nIR light at a working frequency of 1 Hz and irradiated from the side of the PDMS/MNPs layer at an intensity of 15 W/cm^2^, was applied to the end of the soft bilayer away from the cell-cultured regions. In this case, the soft bilayer temperature change was lower than its culturing circumstance temperature, i.e., 37 °C, as shown in Figure 6a. The light spot radius was almost 1.5 mm. We analyzed the maximum temperature distribution along the soft bilayer surface. Infrared images were extracted and compared to analyze the maximum temperature distribution along the soft bilayer surface. It was noticed that the heat region only extended along the length direction while remaining approximately constant along the width direction. This caused the heat region in infrared images to be restricted to almost the same size, i.e., 3 mm in the vertical dimension, as shown in Figure 6b. Upon these analyses, we could conclude that the heated region in the lateral dimension can only reach as large as 4 mm, which implied that the heated region was nearly restricted in the area illuminated by the nIR light. Given that only the MNK-45 cells attached at the first free end of the soft bilayer (i.e., 7–12 mm) were researched, the photothermal effect on the cell viability can be excluded for follow-up quantification. Then, the soft bilayer with cultivated cells was placed back into the incubator and cultured for a further 24–72 h.

**Figure 7:**
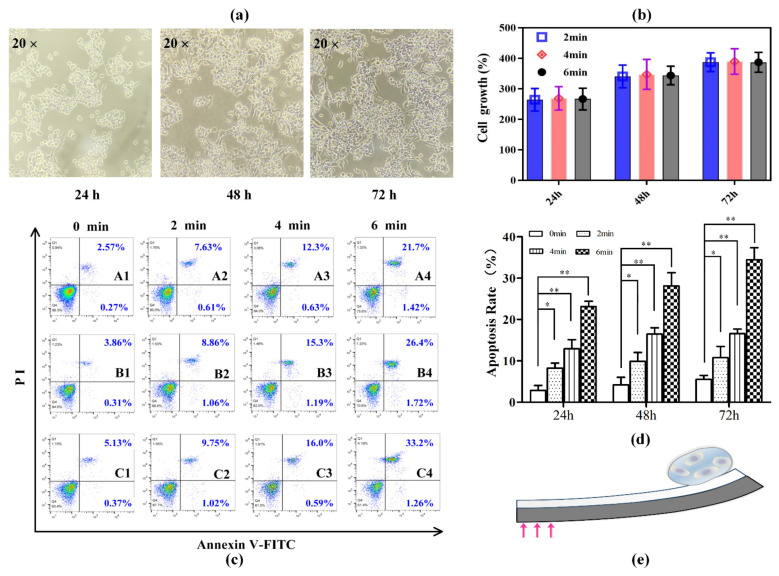
Photomechanical actuation induced MNK-45 cell mechanical responses. (**a**) Normal MKN-45 cell growth morphologies without mechanical stimulation after being cultured for 24, 48, and 72 h. (**b**) Growth rates of normal MKN-45 cells without mechanical stimulation. They were set as the control group. (**c**) Flow cytometry analysis of apoptosis with annexin-V/PI staining. A1–A4: MKN-45 cells were mechanically stimulated with 0, 2, 4, and 6 min for 24 h. B1–B4: MKN-45 cells were treated with 0, 2, 4, and 6 min for 48 h. C1–C4: MKN-45 cells were treated with 0, 2, 4, and 6 min for 72 h. Cells stained with PI were considered as dead cells. The lower left quadrant (annexin V and PI negative) represents viable cells; the lower right quadrant (annexin V positive and PI negative) represents apoptotic cells in the early stage; The upper right quadrant (annexin V and PI positive) represents late apoptotic or necrotic cells; the upper left quadrant (annexin V negative and PI positive) represents necrotic cells or cellular debris. Numbers refer to the percentage of annexin-V and/or PI-labeled cells. (**d**) Total apoptosis rate (early apoptosis rate + late apoptosis rate) comparisons between experimental groups. (* *p* ≤ 0.05, ** *p* ≤ 0.01). (**e**) Photomechanical-actuation-induced mechanical force loading diagram on cells in vitro.

In our cell mechanical force loading experiments, soft-bilayer-cultured normal MKN-45 cells that were not mechanically actuated were set as the control group. Soft bilayers that were actuated by 2, 4, and 6 min were set as the experimental group. Commercial cell counting boards recording cell growth states and flow cytometry characterizing apoptosis rates were conducted for the control group and experimental group, respectively. As shown in Figure 7a, normal MNK-45 cells attached to soft bilayers of the control group were observed under an optical microscope to record the cell growth states. It was noticed that most of the cells were suspended in the culture medium after being cultured for 24 h, which showed clear outlines and were translucent. As time passed, the number of cells increased, and the cell shape gradually became spindle. Cell counting results displayed in Figure 7b also verified the normal MNK-45 cell growth tendency. These results helped exclude the biological incompatibility of our utilized materials for fabricating soft bilayers. Meanwhile, the Annexin V-binding assay was used to quantitatively analyze the effects of mechanical force loading duration on MKN-45 cells. As presented in Figure 7c,d, mechanical force loading conditions could trigger apoptosis of the cells in a stimulation duration time-dependent manner. After 24 h, the mechanical stimulation led to the cell apoptotic rates of 8.24% (stimulated by 2 min), 12.93% (stimulated by 4 min), and 23.12% (stimulated by 6 min), far higher than the control group of 2.84% (0 min, without mechanical stimulation). Particularly, cell apoptotic rates were positively related to the duration time, which was attributed to the fact that longer mechanical force loading duration time enabled higher mechanical force accumulation to affect cell growth and viability. Similarly, in the case of 6 min mechanical force loading, the percentage of apoptotic cells increased up to 28.12% and 34.46% from 4.17% and 5.5% of the control, respectively. These findings provide a foundation for future research into the study of cell differentiation and mechanotransduction. The photomechanical soft bilayer platform proposed in this study, as shown in Figure 7e, may be useful for application of extracellular mechanical forces on cells.

## 4. Conclusions

In conclusion, we produced a novel in vitro mechanical loading tool using a soft and photoresponsive material with a bilayer structure to study mechanotransduction. Due to the photothermal effect of MNPs and the variations in the coefficient of thermal expansion of the two thin layers attributed to MNP incorporation, the soft bilayer demonstrated photomechanical bending under nIR light irradiation with a fast and reversible two-step bending process, which involved fast backlash deflection as the nIR light was switched on and off, and the bilayer bending process. Based on the examination of the velocity and acceleration of the bending process, we determined that the sudden and rapid variations in acceleration made it feasible to load the cells with mechanical forces. In addition, the mechanical force loading tool could qualify temporal and spatial modulations and control and adjust photomechanical deflections due to the controllability and modulation of the nIR source. The movements and oscillations demonstrate the photomechanical bilayer working as an in vitro force loading mechanism. With the gastric gland carcinoma cell line MKN-45 a research subject, it was shown that mechanical force loading conditions could trigger apoptosis of the cells in a stimulation duration time-dependent manner. Cell apoptotic rates were positively related to the duration time, which was attributed to the fact that longer mechanical force loading duration time enabled higher mechanical force accumulation to affect cell growth and viability. Similarly, in the case of 4 and 6 min mechanical force loading, the percentage of apoptotic cells increased up to 28.12% and 34.46% from 4.17% and 5.5% of the control, respectively. In the future, it will be convenient to develop a force-loading tool for use with single cells or small colonies of cells due to the ease of fabrication of the bilayer structure using high-precision microfabrication technology. We anticipate that this will be a significant advance in the development of stimuli-responsive materials that are well-adapted to multivariate mechanical stimulation of specific cells.

## Supplementary Materials

The following supporting information can be downloaded at: https://www.mdpi.com/article/10.3390/ma15196869/s1, Video S1: Large amplitude oscillation of PS sphere motion at the time the nIR light was turned on and off, which was complying with the fast bending process of the soft bilayers.
